# Clinical Effectiveness of Negative Pressure Wound Therapy Following Surgical Resection of Sternoclavicular Joint Infection: A Case Report

**DOI:** 10.7759/cureus.815

**Published:** 2016-10-04

**Authors:** Michelle Nguyen, Susan D Moffatt-Bruce, Robert E Merritt, Desmond M D'Souza

**Affiliations:** 1 Department of Surgery, The Ohio State University Medical Center

**Keywords:** sternoclavicular joint infection, negative pressure wound therapy, septic arthritis, sternum, clavicle

## Abstract

Septic arthritis of the sternoclavicular joint (SCJ) is a rare condition accounting for 0.5% of bone and joint infections. The majority of cases require joint resection and advancement flaps to provide coverage to the resulting wound defect. However, in the setting of an infected wound space, surgeons are often inclined to allow wound healing by secondary intention. Negative pressure wound therapy (NPWT) can be an important adjunct to promote and shorten wound healing time following SCJ resection.

## Introduction

Albeit rare, SCJ infection can cause significant morbidity due to serious complications including sepsis and mediastinitis [1]. Select cases can be treated conservatively by aspiration or drainage in conjunction with intravenous antibiotics but the optimal treatment remains surgical with joint resection and wound closure by ipsilateral pectoralis flap advancement. However, flap coverage and primary closure over an infected wound space can lead to surgical site infection and poor wound healing, and patients can be faced with delayed closure by secondary intention. Over the last 20 years, the use of negative pressure wound therapy (NPWT) has increased dramatically to promote healing of acute or chronic wounds [2]. In addition, NPWT has been shown to be effective in the management of sternal wound dehiscence following cardiac surgery [3-4]. To date, few reports have demonstrated clinical effectiveness of NPWT in proximity to the thoracic inlet, specifically following SCJ resection. This case report describes the course of an IV drug abuser with SCJ infection treated successfully with debridement, resection, and NPWT. Informed consent was obtained from the patient for this study.

## Case presentation

**Figure 1:**
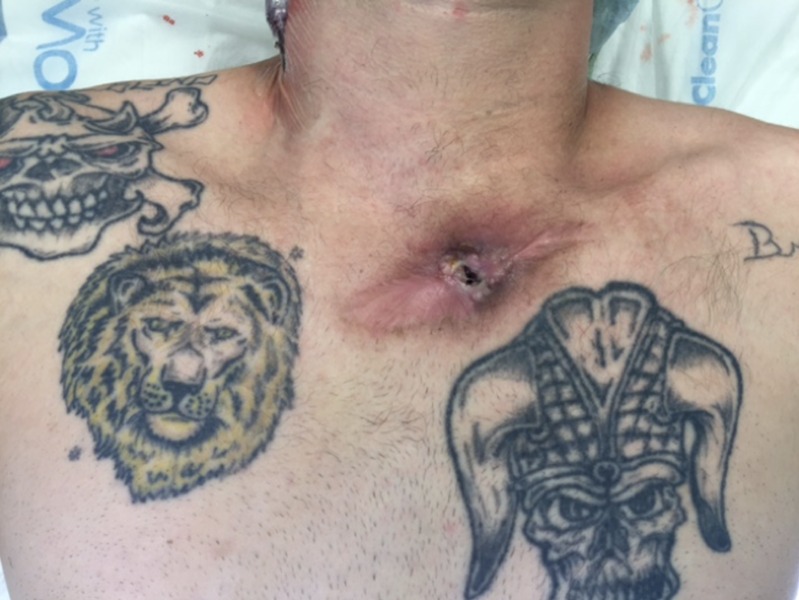
SCJ fistulous tract Physical examination demonstrating a fistulous tract overlying the SCJ with tract epithelialization and scar formation.

**Figure 2:**
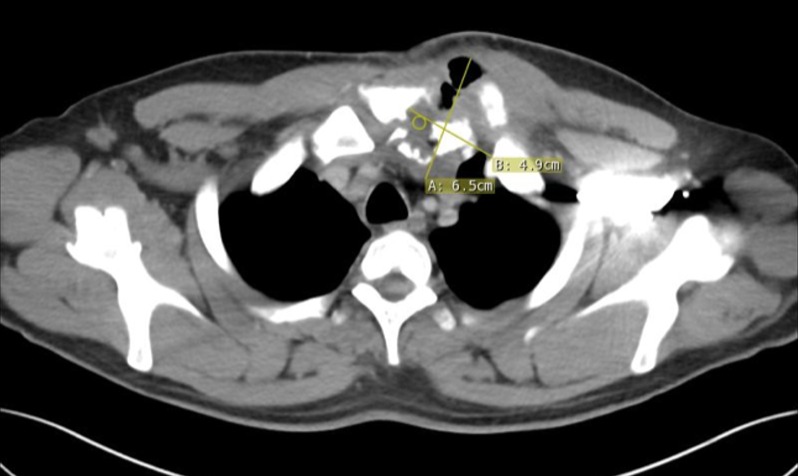
Axial CT Chest An axial CT of the chest with IV Contrast demonstrating destructive changes at the left SCJ and first costochondral junction with fluid collection and air measuring up to 6.5 cm consistent with septic joint and osteomyelitis.

**Figure 3:**
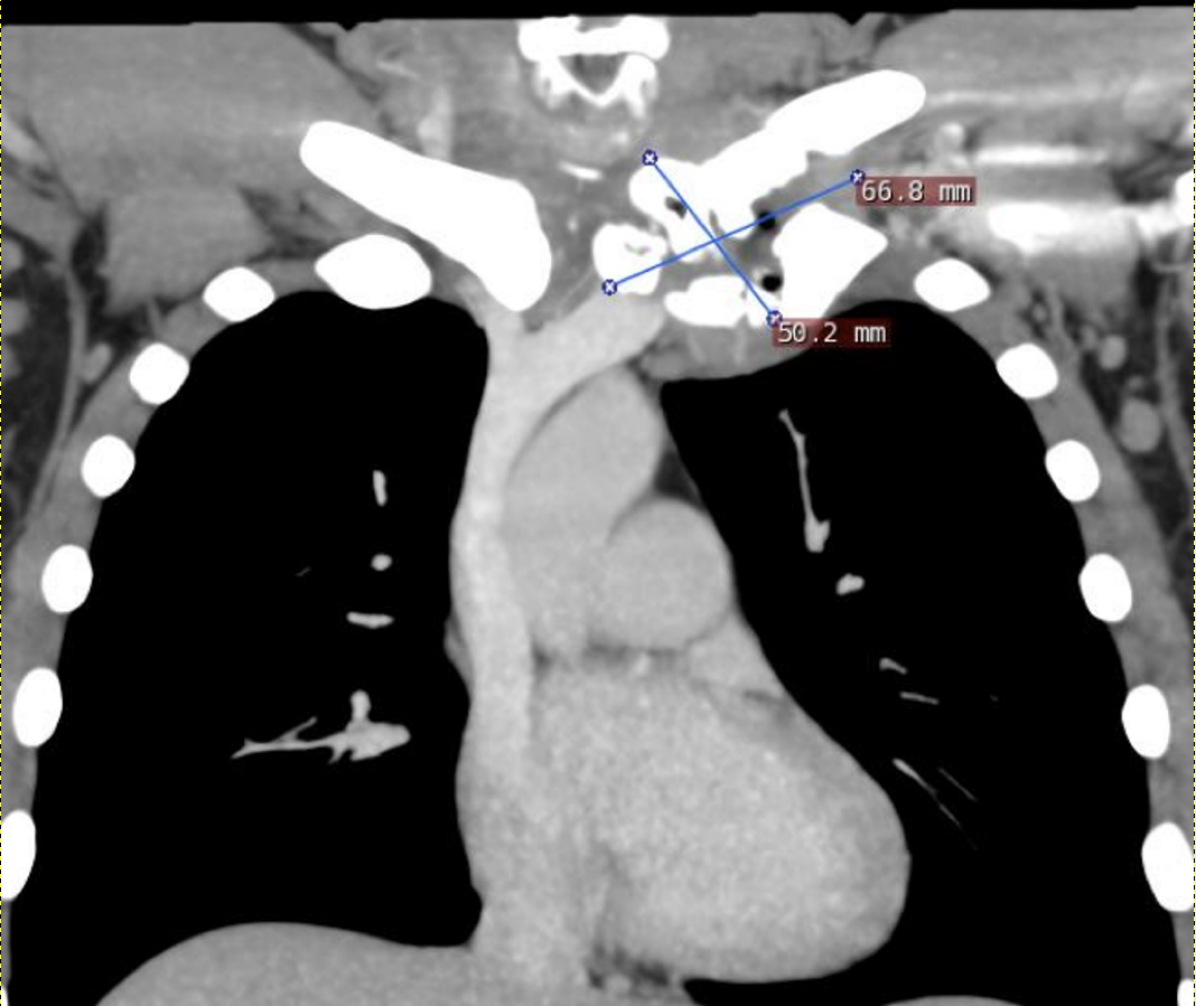
Coronal CT Chest A coronal CT of the chest with IV contrast demonstrating destructive changes at the left SCJ and first costochondral junction with fluid collection and air measuring up to 6.5 cm consistent with septic joint and osteomyelitis.

A 41-year-old Caucasian male with a history of IV drug abuse (IVDA) presented with neck and chest pain in the region of the left sternoclavicular joint. He had previously undergone surgical drainage of an SCJ abscess four months prior to presentation. The patient was afebrile with no leukocytosis on admission. On physical examination, he was found to have a fistulous sinus tract between the joint capsule and the overlying skin with purulent drainage and surrounding erythema (Figure 1). The patient was started on vancomycin and Zosyn for empiric coverage. A computed tomography (CT) of the chest with intravenous contrast demonstrated destructive changes of the manubrium, first left costochondral junction, and head of the clavicle with some periostitis along the distal clavicle. In addition, there was a collection of fluid and air measuring approximately 6.5 x 4.9 cm with superior subluxation of the left SCJ (Figures 2-3).

**Figure 4:**
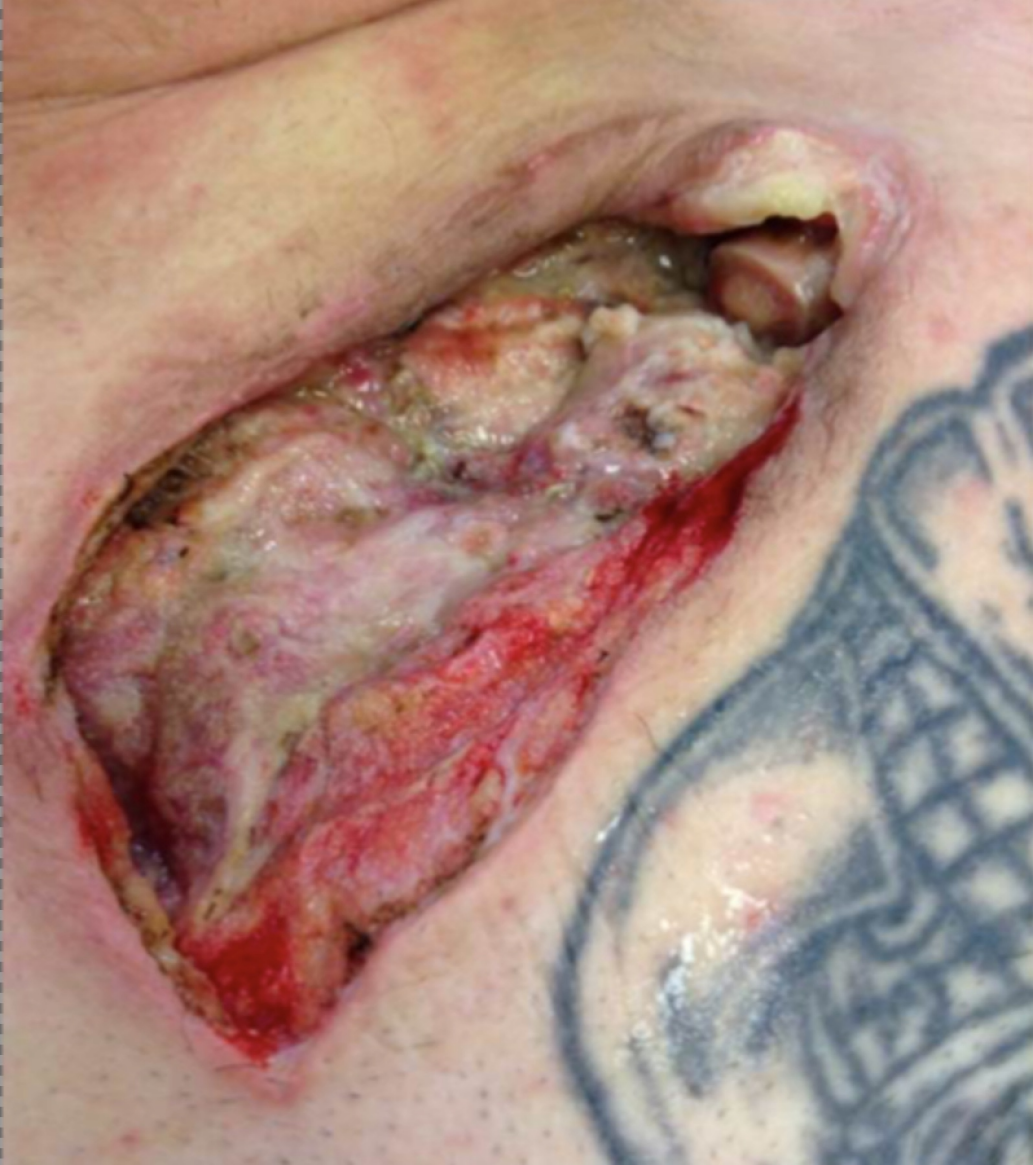
Wound on POD#8 Wound base with fibrinous tissue, peripheral granulation, and exposed distal clavicle.

**Figure 5:**
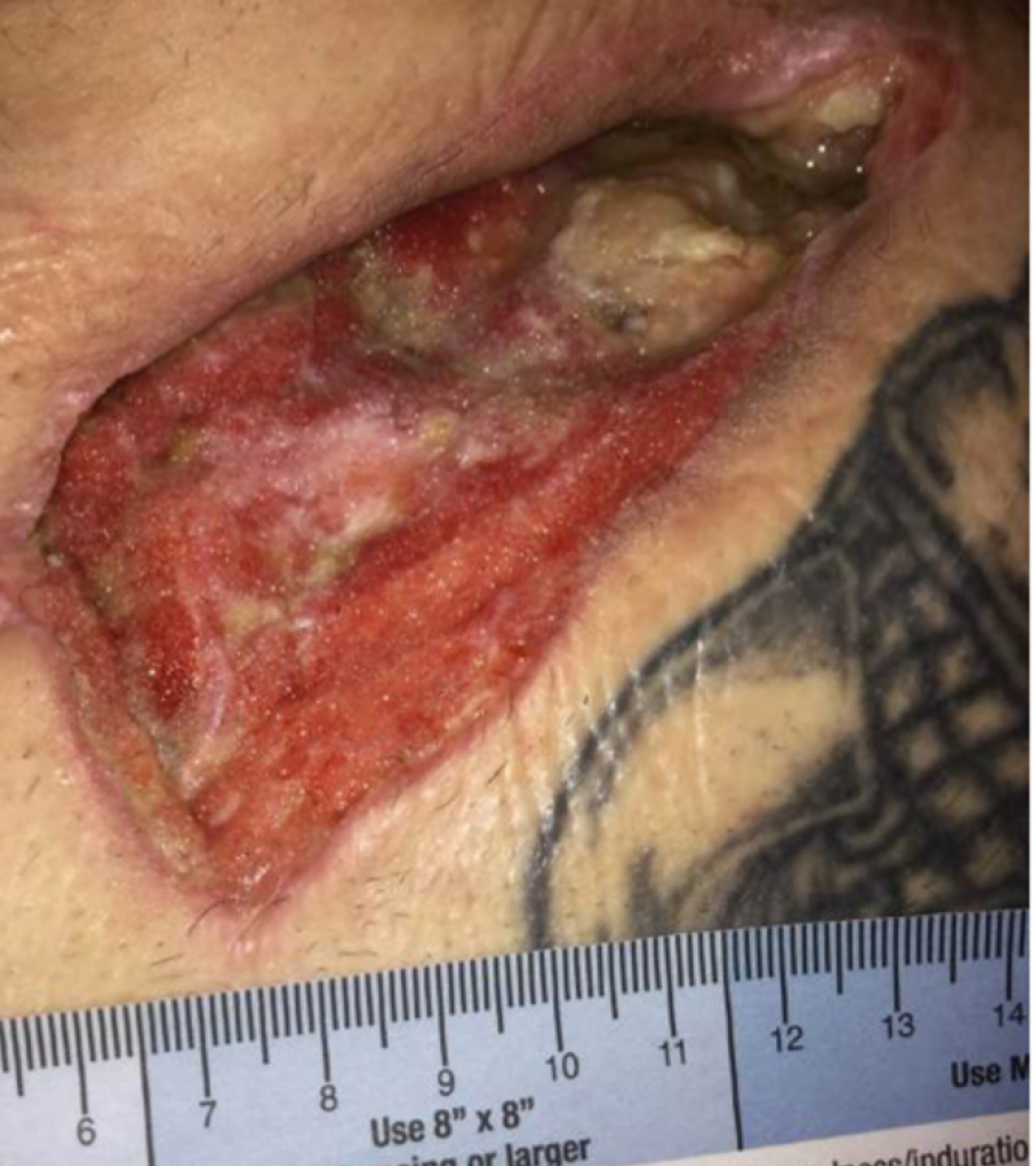
Wound on POD#15 Wound with decreased fibrinous tissue overlying wound base, >80% of wound base granulation, and progression in peripheral epithelialization.

**Figure 6:**
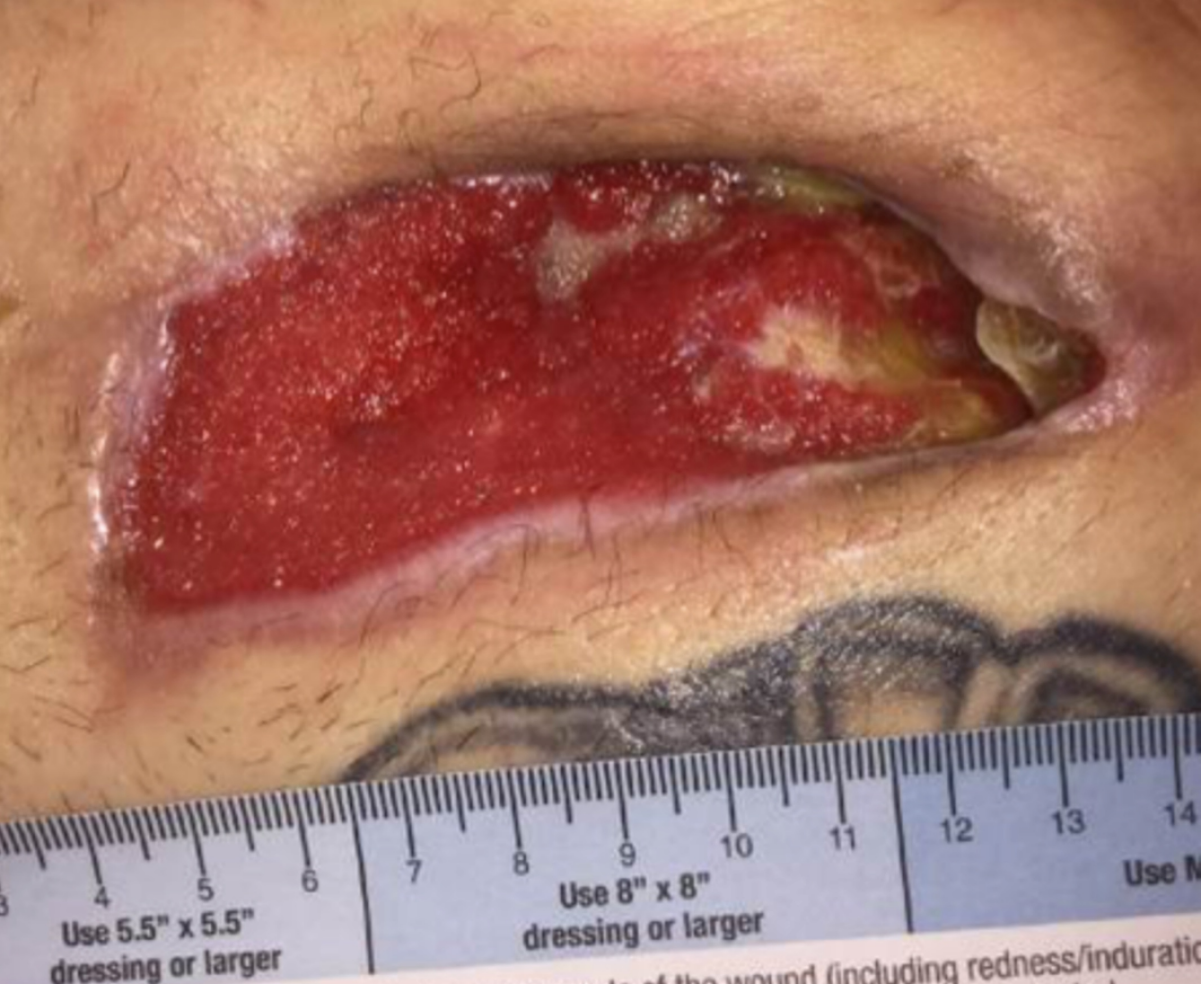
Wound on POD#38 Wound contraction with progressive epithelialization.

**Figure 7:**
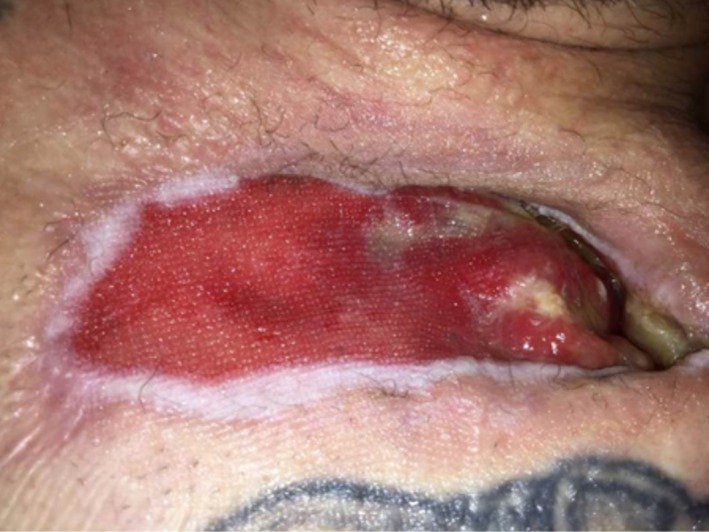
Wound on POD#41 Ongoing wound base granulation, peripheral epithelialization, and peripheral scarring.

He was taken to the operating room for resection of the infected sternoclavicular joint. Exposure was obtained using a curvilinear, inverted L-incision over the medial third of the clavicle extending over the manubrium. The fistula tract was completely excised. The medial third of the clavicle was divided using an oscillating saw. The clavicle completely disarticulated from the left SCJ due to underlying infection. The first rib was spared from infection. The area was then debrided down to healthy tissue and pulse lavage was performed using three liters of bacitracin irrigation. A negative pressure wound (NPW) vacuum (Acelity V.A.C. Ulta™ Negative Pressure Wound Therapy System, San Antonio, TX) was applied to a surface area of 15 square centimeters and connected to a -75 mmHg continuous pressure. Joint cultures grew Corynebacterium and the patient was switched to a six-week course of daptomycin. His wound vacuum-assisted closure (VAC) was changed on postoperative day (POD) one with no evidence of bleeding or continued purulent drainage. The patient was managed on a routine Monday, Wednesday, Friday schedule for NPW VAC change with progressive wound contraction and granulation (Figures 4-7). His hospital course was complicated by acute and chronic pain and a peripherally inserted central catheter (PICC) line deep vein thrombosis (DVT) requiring management with Lovenox and Coumadin. He was eventually discharged on POD#30.

## Discussion

Septic arthritis of the SCJ commonly occurs in the setting of IVDA, diabetes mellitus, immunocompromised state, or existing primary infection at another site or by infected central venous catheter [1]. Many of these risk factors place patients at risk for poor wound healing which may prohibit flap healing. In these patients, closure by secondary intention with the use of NPWT may provide adequate wound epithelialization and granulation. To date, there are limited case series demonstrating the application of NPWT usage in proximity to the thoracic inlet. The area beneath the SCJ contains important neurovascular structures including the subclavian vein and artery, innominate artery, internal mammary artery, and brachial plexus. These structures pose potential for bleeding and nerve damage with the usage of overlying NPWT. Similar anatomical concerns exist in the groin area following vascular grafting; however, several studies and case series have demonstrated the efficacy of applying NPWT to the groin for management of vascular graft infection with low complication rates [5-6]. Our case report demonstrates the efficacy and safety of NPWT in promoting wound epithelialization, contraction, and granulation following SCJ resection. The optimal negative pressure setting over the thoracic inlet is unknown and likely varies according to wound characterization (surface area, edema, fibrosis). We have found that -75 mmHg provides adequate negative pressure without inadvertent vacuum-induced injury to underlying vascular structures. Further studies comparing -100 and -125 mmHg negative pressure provide opportunity for future direction.

## Conclusions

Negative pressure wound therapy is a safe and effective alternative to muscle flap coverage and primary closure following sternoclavicular joint resection in patients who are at risk for poor wound healing.
